# Internal Iliac Artery Ligation in Obstetrics and Gynecology: Surgical Anatomy and Surgical Considerations

**DOI:** 10.3390/clinpract14010005

**Published:** 2023-12-27

**Authors:** Stoyan Kostov, Yavor Kornovski, Rafał Watrowski, Stanislav Slavchev, Yonka Ivanova, Angel Yordanov

**Affiliations:** 1 Research Institute, Medical University Pleven, 5800 Pleven, Bulgaria; drstoqn.kostov@gmail.com; 2 Department of Gynecology, Hospital “Saint Anna”, Medical University, 9002 Varna, Bulgariast_slavchev@abv.bg (S.S.); yonka.ivanova@abv.bg (Y.I.); 3 Department of Obstetrics and Gynecology, Helios Hospital Müllheim, 79379 Müllheim, Germany; rafal.watrowski@gmx.at; 4 Faculty Associate, Medical Center, University of Freiburg, 79106 Freiburg, Germany; 5 Department of Gynecologic Oncology, Medical University Pleven, 5800 Pleven, Bulgaria

**Keywords:** anatomy, obstetrics and gynecology, internal iliac artery ligation, step-by-step, anastomoses, complications

## Abstract

The internal iliac artery (IIA) is the main arterial vessel of the pelvis. It supplies the pelvic viscera, pelvic walls, perineum, and gluteal region. In cases of severe obstetrical or gynecologic hemorrhage, IIA ligation can be a lifesaving procedure. Regrettably, IIA ligation has not gained widespread popularity, primarily due to limited surgical training and concerns regarding possible complications, including buttock claudication, impotence, and urinary bladder and rectum necroses. Nowadays, selective arterial embolization or temporary balloon occlusion are increasingly utilized alternatives, which can be applied preoperatively or intraoperatively for threatening severe genital or pelvic bleeding. However, IIA ligation retains its relevance, as the previously described procedures are not always available and have limitations. This article provides a step-by-step guide to the IIA ligation procedure and its possible complications. It also includes a detailed description of the anatomy of the IIA and pelvic arterial anastomoses. This review highlights the importance of a thorough understanding of pelvic anatomy as a prerequisite for safe IIA ligation and posits that training in this procedure should be an integral part of obstetrics and gynecology curricula.

## 1. Introduction

The internal iliac artery (IIA), previously referred to the hypogastric artery, is the primary arterial vessel of the pelvis. It supplies the pelvic viscera, pelvic walls, perineum, and the gluteal region. The blood supply to the uterus comes from some of the IIA divisions and their anastomoses [1,2]. 

IIA ligation is a potentially lifesaving procedure in cases of severe bleeding in obstetrics and gynecology [3,4,5,6,7]. In 1894, Howard Kelly was the first to introduce this procedure. He ligated both internal iliac arteries and ovarian arteries to control pelvic bleeding when he failed to perform a hysterectomy for patients with advanced cervical cancer [4]. Immediately after the procedure, he observed the cessation of the pelvic hemorrhage and regression of the carcinoma in the following months [4,5]. Hence, the indications of IIA ligation have expanded [3]. Although the procedure has never been widely practiced, it remains one of the treatment methods to manage profuse pelvic hemorrhage in obstetrics [7]. Surgeons are often reluctant to perform the procedure due to concerns about potential complications, such as buttock claudication, impotence, and urinary bladder and rectum necroses [1,5,7]. Contemporary practice increasingly favors alternatives such as selective arterial embolization or temporary balloon occlusion, which can be employed preoperatively or intraoperatively in cases of severe genital or pelvic bleeding [8,9]. However, IIA ligation is still carried out, as the previously described procedures have limitations [10]. Nevertheless, IIA ligation is rarely described in textbooks and surgeons may be hesitant to perform IIA ligation due to a lack of sufficient experience [7,11]. Therefore, the article aims to emphasize the surgical anatomy of the IIA and collateral pelvic vessels, describe the technical steps and surgical considerations of IIA ligation, and provide a comprehensive discussion of its indications and complications.

## 2. Methodology

The review process and the consensus between authors were conducted between January 2023 and July 2023. A comprehensive literature search of scientific articles (studies written in English, French, and German) associated with IIA anatomy, indications, surgical steps during IIA ligation, and perioperative complications due to the procedure was performed. A computer-based extensive review of the MEDLINE, PubMed, EMBASE, and SciSearch databases was performed. The following keywords using Medical Subject Headings (Mesh) terms were used: “internal iliac artery”, “hypogastric artery” “internal iliac artery ligation”, “ internal iliac artery embolization”, “surgical steps”, “complications”, “posterior branch”, “indications”, “haemodynamics”, “anastomoses”. References from the investigated articles were scanned for identification of other related studies. Anatomical textbooks and old, fundamental articles with significant contributions to the field of internal iliac artery ligations have also been used in the present narrative review article. Further information from figures, surgical procedures, and cadaveric dissections performed by the authors was incorporated into the text. 

## 3. Internal Iliac Artery Anatomy

### 3.1. Level of the IIA Origin

The IIA originates from the common iliac artery (CIA) anterior to the sacroiliac joint. Generally, the level of the IIA’s origin is situated at the L5–S1 intervertebral disc [12]. A study involving 150 cadavers identified the bifurcation point of the CIA at the level between L5 and S1 in 82% of cases and at the level of L5 in 15% of cases [13]. Another study, including 60 bisected human cadaveric pelvises, demonstrated that the origin of IIA was at the level of the S1 vertebra in 35 cases (58.3%), at the level of L5–S1 in 24 cases (40%), and in 1 case (1.7%), at the level of L5 [14]. Similarly, in a study involving 50 bisected human cadaveric pelvises, the origin of the IIA was observed at the level of the S1 vertebra in 36 cases (72%), at the level of L5–S1 in 12 cases (24%), and at the level of L5 in 2 specimens (4%) [15]. In the study by Sakthivelavan et al. involving 116 pelvic halves from 58 embalmed cadavers, the origin of the IIA was found at the level of the lumbosacral transition in 94 specimens (81%) and above that level in 22 specimens (19%) [16].

### 3.2. Level of Termination of IIA Trunks

The IIA runs posteriorly to the superior border of the greater sciatic foramen, where it separates into two divisions—anterior and posterior [17]. Mamatha et al. reported the termination of IIA divisions above the level of the greater sciatic notch in 98% of specimens and below it in 2% [15]. A study by Sakthivelavan et al. showed that the IIA originated at the level of the superior border of the greater sciatic notch in 65.5% of cases, and at various positions between lumbosacral transition and the greater sciatic foramen in 34.5% of cases [16]. Naveen observed the termination of the IIA division above the level of the greater sciatic notch in 100% of specimens [14]. Cases have been reported where the posterior division of IIA is absent, or divisions of IIA arise without dividing into anterior and posterior trunks [8,16].

### 3.3. Length of IIA

Knowledge of the length of IIA is essential while performing its ligation. According to Gray’s *Anatomy*, the length of the IIA is approximately 4 cm [1]. The right IIA is frequently longer than the left IIA [13]. Bleich et al. measured the length of the IIA from the CIA bifurcation to the origin of the first posterior division [18]. The authors also measured the length of the IIA together with the cranio-caudal width of the posterior division. Bleich’s measurement has clinical significance, as surgeons can estimate the distance at which the posterior division will be spared. [18]. Generally, the length of the IIA is measured from the CIA bifurcation to the bifurcation into two terminal branches [19,20]. Other authors measured the length of the IIA from its origin to the point of bifurcation of the anterior and posterior divisions [21]. The length of the IIA varies among different populations [21]. Cadaveric studies have reported IIA lengths ranging from 0 to 90 mm (Table 1) [13,14,16,18,19,20,21]. However, except for the study of Bleich et al. [18] (only female cadavers were examined), the majority of studies examined the anatomy of IIA in both sexes [14,16,20,21]. 

### 3.4. Variability in the Branching Pattern of the IIA

The anterior and posterior divisions of the IIA typically include the following branches: posterior division (superior gluteal artery, iliolumbar artery, and lateral sacral arteries) and anterior division (umbilical artery, superior vesical artery (it could be two or more originating), obturator artery, uterine artery, vaginal artery, middle rectal artery, internal pudendal artery, inferior gluteal artery) [1,7,11,22]. Some editions of anatomical textbooks suggest that in females, the vaginal artery may replace the inferior vesical artery [1]. The 42nd edition of Gray’s *Anatomy* stated that in females, the vaginal artery sometimes replaces the inferior vesical artery. The latter can originate in close proximity to the origin of the uterine artery, either as a single vaginal artery or multiple vaginal branches [22]. However, it is worth noting that the presence of the inferior vesical artery in the female population exhibits considerable variability in terms of its origin and prevalence. De Treigny et al. reported finding the inferior vesical artery in 47.4% of the examined female cadavers. The artery had different origins, either as a common trunk with the uterine and umbilical artery (33.3%) or emerging directly from the umbilical artery (33.3%), from the uterine artery (22.2%), or from the obturator artery (11.1%). Consequently, the authors concluded that the presence of the inferior vesical artery should not be considered to be confined to the male population alone [23]. Shafiroff reported a 78% prevalence of the inferior vesical artery among 150 specimens (both males and females). The author observed that the artery has many different origins—the umbilical, testicular, internal pudendal, and vaginal arteries [13]. The presence of inferior vesical arteries was reported in another study, which observed the artery flow velocities by Doppler examination in women receiving hormonal replacement therapy [24]. Ercoli et al. also mentioned the presence of inferior vesical arteries in a cadaveric study, which included 30 female cadavers. Authors observed that the inferior vesical and vaginal arteries are part of the tissue that envelops the vesicovaginal ligament, also known as the deep layer of the vesicouterine ligament [25]. Muallem et al. even used the inferior vesical artery as a landmark for the exact anatomical positions of one of the pelvic splanchnic nerves. Moreover, authors believed that the inferior vesical artery plays a very important role in arterial vascularization of the distal ureter [26]. The presence of the inferior vesical artery was mentioned in another study. Authors divided the IIA into visceral divisions and parietal divisions (the iliolumbar, sacral lateral, superior gluteal, inferior gluteal artery, internal pudendal arteries). The aim of the study was to investigate the variability in the origin of the uterine artery among one hundred women, who underwent laparoscopic surgery for uterine fibroids. Authors reported four different types of IIA origin. The study found that the uterine artery, middle rectal artery, and the inferior vesical artery originated from the internal pudendal artery (type 2b) in 24.3% of the investigated patients [27]. Authors reported that the inferior vesical artery, the middle rectal artery, and the uterine artery arose in a common trunk, which originated from a larger-than-usual internal pudendal artery [27]. 

It is well established that the IIA exhibits numerous variations in its branching pattern. Therefore, a thorough understanding of the distribution pattern of this artery is essential for safely performing retroperitoneal surgery [12]. 

**Figure 1:**
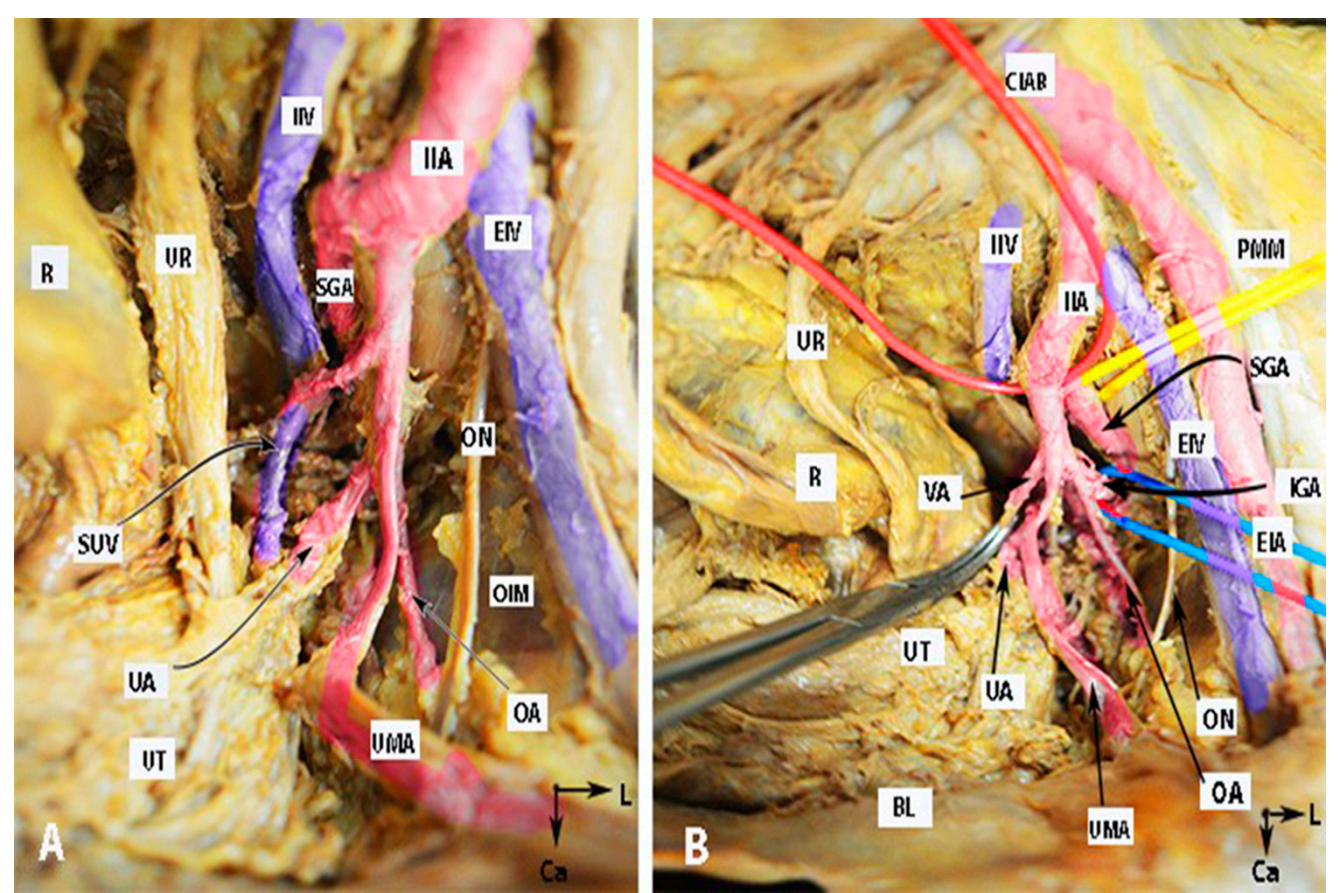
Anatomy of the internal iliac artery ((**A**,**B**)—embalmed female cadaver; author’s own material). (**A**,**B**) show the left side of the pelvis of the same cadaver. The posterior branch of the IIA has a lateral course (superior gluteal artery) in the pelvic sidewall. The middle rectal artery and iliolumbar artery were cut during dissection. EIA—external iliac artery; IIA—internal iliac artery; EIV—external iliac vein; ON—obturator nerve; OIM—obturator internus muscle; SGA—superior gluteal artery; IGA—inferior gluteal artery; UR—ureter; SUV—superficial uterine vein; OA—obturator artery; UMA—umbilical artery; UA—uterine artery; UT—uterus; PMM—psoas major muscle; IIV—internal iliac vein; VA—vaginal artery; CIAB—common iliac artery bifurcation; BL—bladder; R—rectum; CA—caudal; L—left.

**Figure 2:**
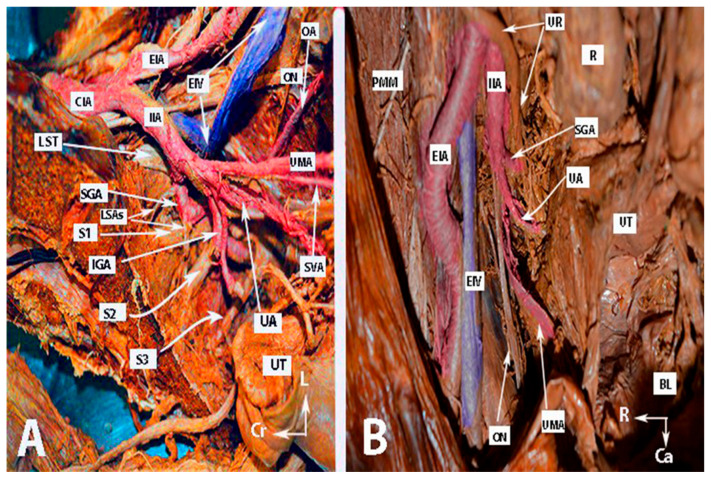
Anatomy of the internal iliac artery ((**A**,**B**)—embalmed female cadavers; author’s own material). (**A**,**B**) show the left and right pelvic sidewall in two different female cadavers. The posterior branch of the internal iliac artery (superior gluteal artery) has a medial course in both figures. (**A**)—The sacral plexus and its relation to the divisions of the internal iliac artery are clearly highlighted. The superior gluteal artery runs between the lumbosacral trunk and the anterior ramus of the first sacral nerve and leaves the pelvis through the suprapiriform foramen. The inferior gluteal artery is located between the anterior rami of the second and third sacral nerves and leaves the pelvis through the infrapiriform foramen. (**B**)—The course of the uterine artery ventral to the ureter can be clearly seen. EIA—external iliac artery; IIA—internal iliac artery; EIV—external iliac vein; ON—obturator nerve; SGA—superior gluteal artery; IGA—inferior gluteal artery; UR—ureter; SUV—superficial uterine vein; SVA—superior vesical artery; LSAs—lateral sacral arteries; OA—obturator artery; UMA—umbilical artery; UA—uterine artery; PMM—psoas major muscle; IIV—internal iliac vein; VA—vaginal artery; CIAB—common iliac artery bifurcation; BL—bladder; UT—uterus; R—rectum; LST—lumbosacral trunk; S1, S2, S3—anterior rami of the sacral spinal nerves; Ca—caudal; L—left; Cr—cranial.

**Figure 3:**
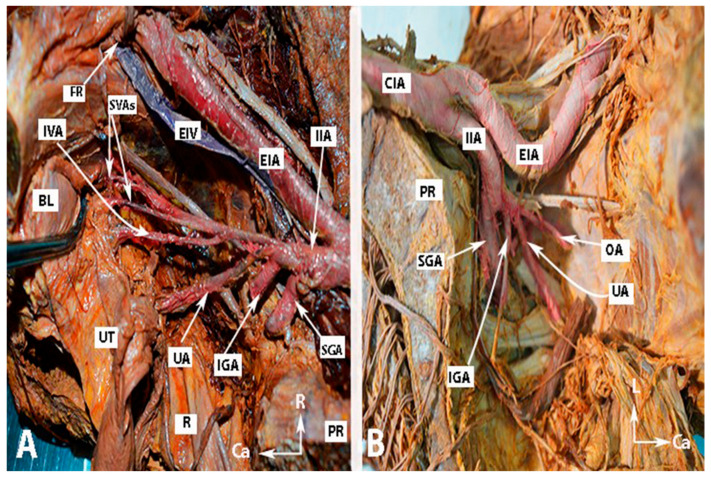
Anatomy of the internal iliac artery of (**A**,**B**) embalmed female cadavers; author’s own material. (**A**,**B**) show the right and left pelvic sidewalls in two different female cadavers. A—Presence of the inferior vesical artery in a female. B—Anomalous shape and course of the internal and external iliac arteries. The internal iliac artery follows a direct caudal course. EIA—external iliac artery; EIV—external iliac vein; IIA—internal iliac artery; FR—femoral ring; IVA—inferior vesical artery; SVAs—superior vesical arteries; IGA—inferior gluteal artery; SGA—superior gluteal artery; UA—uterine artery; OA—obturator artery; PR—promontory; UT—uterus; R—rectum; BL—bladder; R—right; Ca—caudal; L—left.

Many authors have proposed various concepts for classifying the terminal divisions of the IIA [20,28,29]. Generally, surgeons use the Adachi classifications system, which has been the standard for many years [15,16,19,20]. Adachi defined the branching of the IIA based on its four major parietal divisions: the umbilical, superior gluteal, inferior gluteal, and internal pudendal arteries (see Figure 1, Figure 2 and Figure 3) [12,19].

The Adachi classification system includes the following types:

Type 1: The superior gluteal artery (SGA) originates separately from the IIA, while the inferior gluteal (IGA) and internal pudendal artery (IPA) share a common trunk. Subtypes 1a and 1b depend on whether the bifurcation of the IGA and IPA occur within or below the pelvis, respectively.

Type 2: The IPA originates independently from the IIA, while the SGA and the IGA share a common trunk. Subtypes 2a and 2b represent whether the bifurcation of the SGA and IGA arise within or below the pelvis, respectively.

Type 3: The SGA, IGA, and IPA originate independently from the IIA, with the IPA being the terminal branch.

Type 4: The SGA, IGA, and IPA have a common trunk. Classification in subtypes 4a and 4b depends on whether the SGA or IPA is the initial vessel emerging from the common trunk. For instance, in 4a, the SGA is the initial vessel coming from the common trunk before bifurcating into the other two divisions—the SGA and IGA. In type 4b, the IPA is the first to come from the common trunk, which then splits into the SGA and IGA. 

Type 5: The IGA originates independently from the IIA, while the SGA and IGA share a common trunk [19].

Surgeons should also be familiar with the anatomy of the internal iliac vein (IIV), which follows a similar course to its arterial counterpart, ascending posteromedially to the IIA. The IIV drains into the ipsilateral external iliac vein [1].

For visual reference, the anatomy of the IIA and some anatomical differences are illustrated in Figure 1, Figure 2 and Figure 3. The iliolumbar artery and middle rectal artery are not shown in the figures.

## 4. Indications for IIA Ligation

The indications for IIA ligation can be prophylactic or therapeutic in both gynecological and obstetrical situations [5,30,31,32]. The need for IIA ligation arises from increased intrabdominal bleeding recognized before, during, or after surgery. “Increased intra-abdominal bleeding” is defined according to Watrowski et al. as “any bleeding which could not be sufficiently managed with conventional hemostatic maneuvers (sutures, clips etc.), resulting in measured or expected blood loss of more than 1 L, or any intra-abdominal bleeding leading to cardiovascular instability, or requiring transfusion of blood products” [33]. The prerequisites for IIA ligation is that the source of bleeding lies or is suspected in the blood flow area of the IIA, and there is not enough time for conventional surgical maneuvers or hemostatic agents. Typical indications for IIA ligation are listed below [5,6,7,11,30,31,32,34]:

Obstetrical indications (including early pregnancy):

- Ruptured ectopic non-tubal pregnancy (cervical, interstitial, or in the peritoneum in the rectouterine pouch);
- Severe cervical or uterine injury during surgical abortion;
- Uterine rupture before or during delivery;
- Severe obstetrical lacerations (with or without instrumental delivery) extending to the uterine cervix or parametrium;
- Placenta previa;
- Placental abruption;
- Placenta accreta spectrum;
- Postpartal retroperitoneal hematoma.

Gynecological indications:

- Uncontrollable hemorrhage from advanced uterine, vaginal, or vulvar cancer;
- Prophylactic ligation during oncogynecological procedures with expected profuse bleeding (e.g., pelvic exenteration);
- Pelvic hemorrhage or massive retroperitoneal hematoma due to iatrogenic (e.g., trocar insertion) or traumatic (gunshot or fracture) injury;
- Massive pelvic hemorrhage or retroperitoneal pelvic hematoma due to primary or secondary coagulation disorders (e.g., with no visible bleeding);
- Actual or possible hemorrhage in patients who refuse blood transfusion (e.g., Jehovah’s Witnesses).

## 5. Why Is Internal Iliac Artery Ligation Still a Viable Option?

Selective arterial embolization (SAE) or temporary balloon occlusion (TBO) represent less invasive alternatives for hemorrhage control [6,8,9]. These procedures can be performed prophylactically and are the primary methods in facilities where they are available [8,9]. However, their utility in cases of unexpected extensive bleeding is limited [7]. Additionally, SAE and TBO come with several limitations [6,10,35]:

- Specialized equipment and expertise are prerequisites.
- They are less effective in traumatic patients or instances of unforeseen bleeding.
- Patients must be hemodynamically stable without coagulation disorders.
- The procedures typically require at least one hour.
- Access to the radiology department may necessitate relocating to another building.
- SAE or TBO procedures are infrequently performed in low- or middle-income countries.
- SAE has specific contraindications, including uterine rupture and eversion (which should be managed surgically), arterial anomalies, coagulopathy, impaired renal function, and contrast material allergies.

A study comparing complication rates between IIA ligation and embolization in vascular surgery, oncology, and trauma patients revealed that embolization was associated with an increased risk of complications compared to IIA ligation (16.7% vs. 4.1%). The authors concluded that IIA interruption (through embolization or ligation) is safe for young patients in obstetrics and gynecology. However, they emphasized a preference for IIA ligation [2]. Consequently, surgical ligation of the IIA remains a part of the majority of guidelines for managing gynecological and obstetric hemorrhage [35,36].

## 6. Surgical Technique of IIA Ligation

Ligations of the IIA can be performed through an open surgery or minimally invasive approach, and either transperitoneally or extraperitoneally [3,7,10,11]. In this article, we describe the transperitoneal approach. 

Step 1. Approach to the abdomen (laparotomy or laparoscopy)

In cases requiring elective IIA ligation, access to the abdominal cavity is created. Depending on clinical scenarios and surgeon expertise, a midline or transverse laparotomy or laparoscopy is performed. Midline laparotomy is preferred for planned oncological resections, emergencies with hemorrhage of unknown origin, or surgeries involving the upper abdomen. The IIA itself does not require a conversion from laparoscopy to laparotomy. Surgeons experienced in minimally invasive surgery can successfully perform laparoscopic IIA ligation [37,38,39,40]. 

Step 2. Identification of the common iliac artery bifurcation

Transperitoneal visualization of the CIA bifurcation is essential. In patients with a high body mass index, the following maneuvers can be considered [11]:

- As mentioned earlier, the origin of the IIA is typically found at the level of the promontory.
- Visualize an imaginary bony line that passes through both anterior superior iliac spines.

Step 3. Incision of the parietal peritoneum

When the uterus is present, a horizontal incision should be made starting from the round ligament of the uterus and extending cranially to the level of the CIA. The incision is between the ovarian vessels and the psoas major muscle. The uterus should be retracted opposite to the peritoneal incision. If the uterus has been removed, the incision can start from the external iliac artery up to the CIA bifurcation. Alternatively, an incision slightly lateral to the CIA, extending caudally, can be made to locate the origin of the IIA and external iliac artery [11]. In thin patients with transperitoneally visible ureters, the incision may start laterally and run parallel to the ureter, which remains in the medial fold of the peritoneal incision [31]. 

Step 4. Identification of the ureter 

This step is crucial. The ureter should always be identified and retracted medially from the iliac vessels. The relationship of the ureter to the IIA is shown in Table 2 [1,11]. 

As shown in Table 2 the anatomical relationships between the left and right IIA exhibit subtle differences that have not been commonly discussed in the context of bilateral IIA ligation procedures. These differences arise from the distinct positions of the ureter relative to the CIA bifurcation. According to Luschka’s law, the left ureter typically crosses the iliac artery approximately 1.5 cm below the CIA bifurcation, whereas the right ureter crosses the iliac artery about 1.5 cm above the bifurcation [10,41,42]. Consequently, the ureter enters the pelvis by crossing the CIA on the left side and the external iliac artery on the right side. During dissection for bilateral IIA ligation, surgeons must be aware of this variation to prevent inadvertent injury to the ureter. It may be prudent to place a sling or vessel loop around the identified ureter for added safety. Moreover, surgeons should also be acquainted with potential ureteric variations, such as double ureter, bifid ureter, or ureteral diverticula [11,41,42].

Step 5. Identification of the IIA

After dissection of the areolar connective tissue, the bifurcation of the CIA becomes visible. The dissection should progress in a cranio-caudal direction, closely tracking the course of the CIA to prevent injury to underlying veins. The external iliac artery and IIA should become visible, forming a “ tripod” structure, with the ureter located medially and the external iliac arteries positioned laterally. The structure in the middle should be the IIA. To enhance certainty just before ligation, palpation of the bounding pulse over the arteries is recommended. The IIA typically follows an inferior and medial course, whereas the external iliac artery has a horizontal (slightly superior just before entering the femoral ring) and a lateral course. Additionally, once the psoas major muscle is identified, the external iliac artery can be found over and slightly medial to the muscle [3,7,10,11].

Step 6. Lateral pararectal space development

The boundaries of the lateral pararectal space (Latzko’s space) are, medially, the ureter, laterally the IIA, ventrally the transverse cervical ligament (cardinal ligament of the uterus), dorsally the pelvic surface of the sacrum, caudally the levator ani muscle, and cranially the parietal peritoneum [43]. By dissection of this space, the ureter is additionally retracted medially. In addition, it offers sufficient space for the procedure.

Step 7. Dissection between the IIA and the underlying ipsilateral vein

This step is the most critical part of the procedure since the artery often firmly adheres to the underlying vein. The dissection between the artery and the vein must be meticulous. The internal iliac vein is located below and slightly medial to the IIA. Clearing the adventitia of the artery may help to identify the correct dissection plane [11,44,45,46,47,48]. The artery can be gently elevated without injuring the vein. It is advisable to use blunt right-angled forceps (like Lahey, Mixter forceps or aneurysm needle) and separate the vessels by meticulous dissection using blunt-tip scissors. Opening the instrument tips between the vein and artery in an uncontrolled manner should be avoided [11,31,45]. Fortunately, anatomical variations in the IIA 2–3 cm from its origin are not common. However, surgeons should be aware of internal iliac veins’ variations, which, although not frequent, can include double veins, anastomoses between the external and internal iliac veins, or the absence of the internal iliac vein, replaced by numerous anomalous veins) [12]. 

Step 8. Ligation of the anterior division of the IIA

The instrument should be passed beneath the artery, approximately 3 cm below the bifurcation, from lateral to medial in order to ensure the ligation of only the anterior division and to avoid iatrogenic injury to the external iliac vessels. Identifying the posterior division of the IIA is unnecessary and could even be dangerous, potentially leading to injury of the underlying veins [46,47,48]. The tips of the instrument should be directed slightly toward the IIA and not toward the underlying vein [3,11,13,31,45,46]. It is important that the tips of the forceps point toward the inferior border of the IIA because there is less risk of IIA injury (the artery has stronger walls than the vein). After identifying the instrument’s tips beneath the IIA, a doubled No. 0 or 1.0 absorbable suture is grasped and pulled backward in the same direction [42]. The tips of the instrument should not point to the internal iliac vein when removing the forceps between the artery and vein. The ureter, external iliac vessels, and the IIA should be re-checked just before the suture [46]. The ligatures should be tied firmly in order to avoid transection of the artery [3,11,13,31]. Immediately after suturing the artery, the pulsation of the ipsilateral femoral artery within the inguinal region should be confirmed [47]. It is not necessary to divide the vessel between two ligatures, as doing so could be dangerous and may lead to iatrogenic injury to the underlying vein [5,48]. The procedure may be more challenging on the left side due to the common adhesions of the sigmoid colon or mesocolon to the pelvic sidewall. In such cases, the colon should be dissected at the white line of Toldt [11].

**Figure 4:**
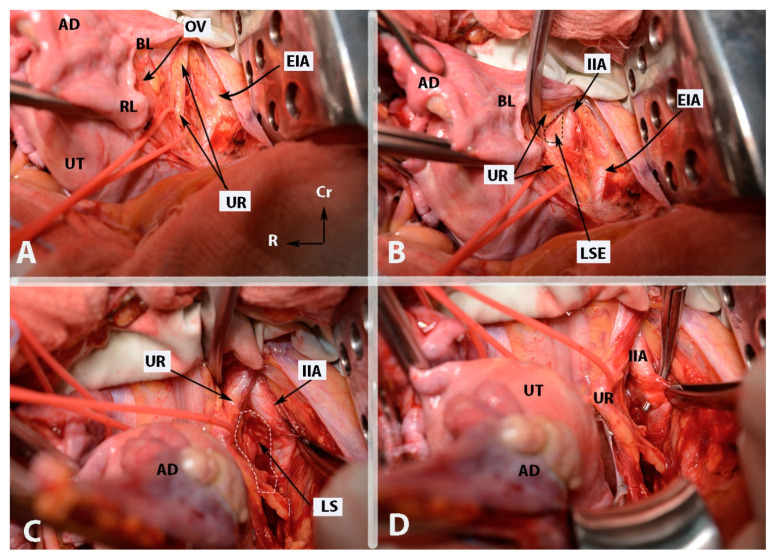
Some surgical steps during internal iliac artery ligation in case wherein the uterus is presented (open surgery (left side)—author’s own material). (**A**)—incision of the parietal peritoneum and identification of the ureter (steps 3 and 4). (**B**)—Identification of the internal iliac artery and entrance point of Latzko’s lateral pararectal space (steps 5 and 6). (**C**)—Development of lateral pararectal space (step 6). (**D**)—Ligation of the internal iliac artery. The posterior division is located just caudal to the surgical clamp. The instrument passes from lateral to medial beneath the artery (step 8). EIA—external iliac artery; OV—ovarian vessels; UR—ureter; UT—uterus; BL—posterior leaf of broad ligament; RL—round ligament (cut); AD—left adnexa; IIA—internal iliac artery; LSE—lateral pararectal space entrance; LS—lateral pararectal space; Cr—cranial; L—left.

**Figure 5:**
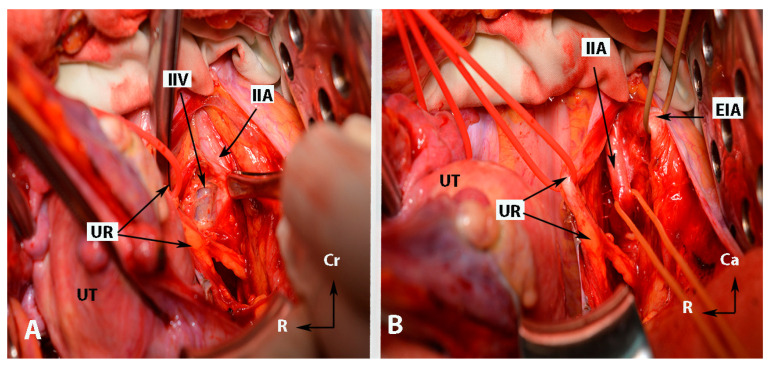
Some surgical steps during internal iliac artery ligation in case wherein the uterus is presented (open surgery (left side)—author’s own material). (**A**)—dissection between the IIA and the underlying ipsilateral vein (step 7). (**B**)—The “ tripod” structure, with the ureter located medially and the external iliac artery positioned laterally. The structure in the middle is the internal iliac artery. EIA—external iliac artery; IIA—internal iliac artery; UR—ureter; UT—uterus; IIV—internal iliac vein; Cr—cranial; R—right.

Surgical steps are depicted in Figure 4 and Figure 5.

## 7. Perioperative Complications

Knowledge of surgical anatomy, differentiated usage of surgical and hemostatic tools, and adherence to basic safety principles are precautions for reducing complications’ incidence and severity. Adverse events following IIA ligation can occur immediately or in the postoperative period. 

Intraoperative complications may include injury to the iliac vessels, ligation of the external or CIA, or damage to the ureter [3,11,45,46]. Surgeons should be familiar with the nerve compartment (the obturator nerve, truncus lumbosacralis, S1–S5 anterior rami of the sacral spinal nerves and the sciatic nerve) of the pelvic sidewall. In rare cases, the obturator nerve, truncus lumbosacralis, or the sciatic nerve may be injured or mistaken for the IIA. The truncus lumbosacralis is located below the vascular compartment (contains the internal iliac vessels and its posterior branches) in the pelvic sidewall, and its identification means that the surgeon performed an unnecessary caudal dissection [11]. 

Postoperative complications primarily result from reduced blood flow and subsequent necrosis of the buttocks, gluteus maximus muscle, and pelvic visceral organs. Moreover, hip claudication, urinary bladder atony, lumbosacral plexopathies, and impotence may also be observed [10,11,49]. Therefore, it is crucial that ligation of the IIA should be performed distal to its posterior division (SGA) [10,11,45,46,47,48,49]. Fortunately, these complications are rare due to the extensive collateral circulation in the pelvis after ligation [10,11,48,49]. 

Andriole and Sugarbaker reported vesical and perineal necrosis after IIA ligation in a patient operated on for recurrent rectal cancer, with a previous history of radiotherapy [50]. Bangal et al. reported ischemic necrosis of the buttock in 1 patient out of 54 women who underwent ligation of the artery due to obstetrics and gynecology complications [51]. Ischemic complications have been also observed after IIA embolization. Gezer and Çakır reported lumbosacral neuropathy and buttock necrosis after IIA embolization in a patient with vaginal bleeding due to locally advanced cervical cancer [52]. Although these complications are rare, they are predominantly observed in older patients with aorto-iliac occlusive disease, a history of previous radiotherapy, insulin-dependent diabetes, end-stage renal disease, or after rectal cancer surgery (during rectal resection, the collaterals from the superior rectal artery are absent) [10,53,54,55,56]. From these studies, it can be concluded that ischemic complications are mostly observed in patients with interrupted vascular flow and pre-existing vascular risk factors [10,53,54,55,56]. It is imperative to mention that ischemic complications are more commonly observed in patients who underwent embolization of the IIA [57,58].

However, Shin et al. reported a case of peripheral nerve ischemia after IIA ligation in an 18-year-old woman at 40 weeks of gestation with preeclampsia. The ligation was performed due to uterus atony and severe postpartal hemorrhage. Authors concluded that risk factors such as preeclampsia and postoperative endometritis could predispose the patient to ischemic complications [56]. The ischemia to the femoral nerve and the sciatic nerve routes could occur in cases of infarction of the terminal branches of the SGA after IIA ligation [56]. Spinal cord ischemia after interruption of the blood flow to the IIA has also been described [53]. 

## 8. Hemodynamics after IIA Ligation

The primary hemodynamic effect following bilateral IIA ligation is the nearly complete elimination of blood pressure in small arteries distal to the ligation, effectively transforming the arterial system into a venous one. As a result, a hemostatic mechanism develops, leading to the formation and retention of clots [44,59,60,61]. The pulse pressure and arterial blood pressure distal to the ligation decrease by 85% (with bilateral IIA ligation) and 25% (bilateral IIA ligation), respectively [59,60,61]. Therefore, bilateral IIA ligation is more effective than unilateral ligation [11]. Unilateral ligation has limited indications such as unilateral retroperitoneal hematoma or unilateral bleeding from the pelvic sidewall [48]. 

## 9. Anastomoses in the Pelvis and Their Relation to IIA Ligation

Anastomoses among the IIA divisions can be categorized into horizontal and vertical types. Horizontal anastomoses are not related to IIA ligation since they occur between the divisions of the left and right IIA. These anastomoses remain intact in cases of unilateral IIA ligation but are eliminated in bilateral ligation. They are between the contralateral branches of the IIA—obturator arteries, inferior/middle rectal arteries, superior/inferior vesical arteries, and lateral sacral arteries [5,6,11,13,49,62]. The contralateral anastomosis between the uterine arteries could be subdivided into three types regarding arterial branch location to the uterine wall—subperitoneal, submucous, and parenchymatous [63,64]. Most horizontal anastomoses between both uterine arteries are microscopic. However, there are also microscopic transverse (horizontal) arterial anastomoses known as Huguier’s circles, which are located near the uterine isthmus [63,64]. The superior vesical artery and the inferior vesical artery anastomose at the level of the ureterovesical junction. The inferior vesical artery supplies the lower wall of the retrovesical ureter at the level of the ureterovesical junction [65]. Surgeons believe that this anastomosis prevents the formation of ureterovaginal fistulas during radical hysterectomy, where the ureter should be entirely mobilized to the level of the urinary bladder [26,65].

Vertical anastomoses, on the other hand, play a significant role in bilateral IIA ligation and can be further divided into three groups [49,50].

The first group is relevant when the uterus is preserved. These anastomoses occur between divisions of the uterine artery and the inferior epigastric artery (at the level of the round ligament of the uterus) or between the uterine artery and the ovarian arteries (at the level of mesosalpinx and mesovarium) [11,63]. The anastomoses between the terminal branches of the ovarian artery and the branches of the uterine artery form a vertical arterial arcade, which is known as utero-ovarian anastomoses. However, the anastomoses are not constant, as studies reported an incidence in from 11 to 51% of cases [64,66,67]. 

**Figure 6:**
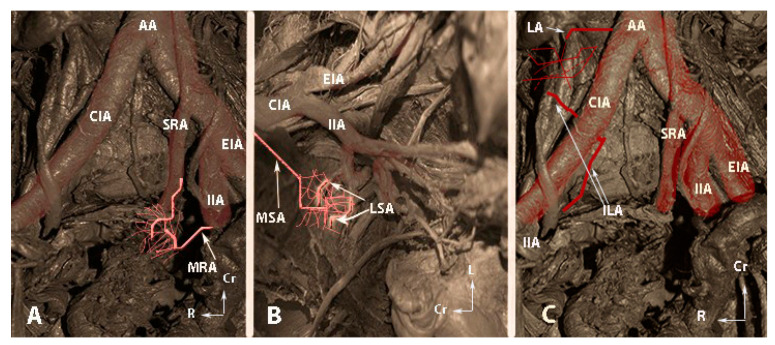
The second group of anastomoses (embalmed cadavers; author’s own material). (**A**)—Anastomoses between the middle rectal artery and the superior rectal artery. (**B**)—Anastomoses between the lateral sacral arteries and the median sacral artery. (**C**)—Anastomoses between the lumbar artery and the iliolumbar artery. AA—abdominal aorta; CIA—common iliac artery, SRA—superior rectal artery; MRA—middle rectal artery; IIA—internal iliac artery; EIA—external iliac artery; MSA—median sacral artery; LSA—lateral sacral artery; LA—fourth lumbar artery; ILA—iliolumbar artery, Cr—cranial; L—left; R—right.

The second group is the most important and plays a crucial role after ligation. It includes anastomoses between the lateral sacral arteries and the median sacral artery, the fourth lumbar artery and the iliolumbar artery, and the middle rectal artery and the superior rectal artery 9 [5,6,11,13]. Burchell and Olson performed pelvic aortograms in 22 patients, of which 19 underwent previous IIA ligation (16 patients had bilateral and 3 unilateral internal iliac artery ligation). The three women without IIA ligation were a control group. Authors found that the anastomoses between the middle rectal artery and the superior rectal artery became active only after IIA ligation above the posterior division [59]. However, the anastomoses between the lateral sacral arteries/median sacral artery and the fourth lumbar artery/iliolumbar artery were active after IIA ligation below the posterior division [59]. The Burchell studies showed that these two anastomoses are the first and main anastomoses after IIA ligation [59,61]. These studies had a fundamental impact, as it was wrongly believed that the main collateral blood supply in the pelvis after IIA ligation arose from the branches of external iliac artery and superior/inferior gluteal arteries [59,60,61]. The second group of anastomoses are shown in Figure 6.

**Figure 7:**
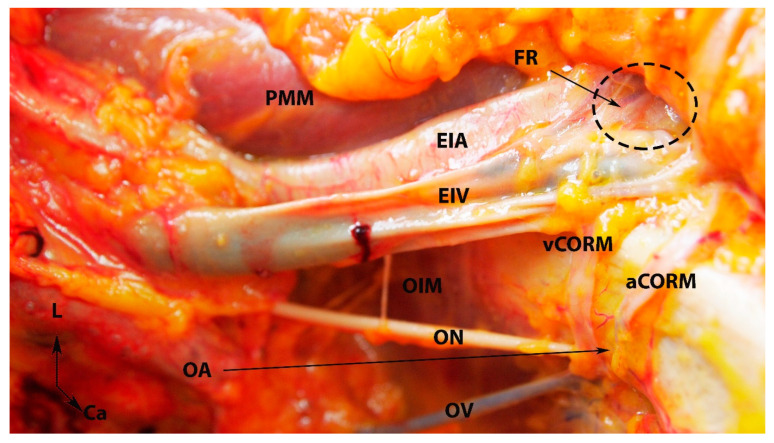
Arterial and venous corona mortis (fresh cadaver; author’s own material). EIA—external iliac artery; EIV—external iliac vein; PMM—psoas major muscle; OA—a proper obturator artery was injured during dissection. There was no accessory or aberrant obturator artery; ON—obturator nerve; OV—obturator vein; OIM—obturator internus muscle; FR—femoral ring; PS—pubic symphysis; aCORM—arterial corona mortis; vCORM—venous corona mortis; L—left; Ca—caudal.

**Figure 8:**
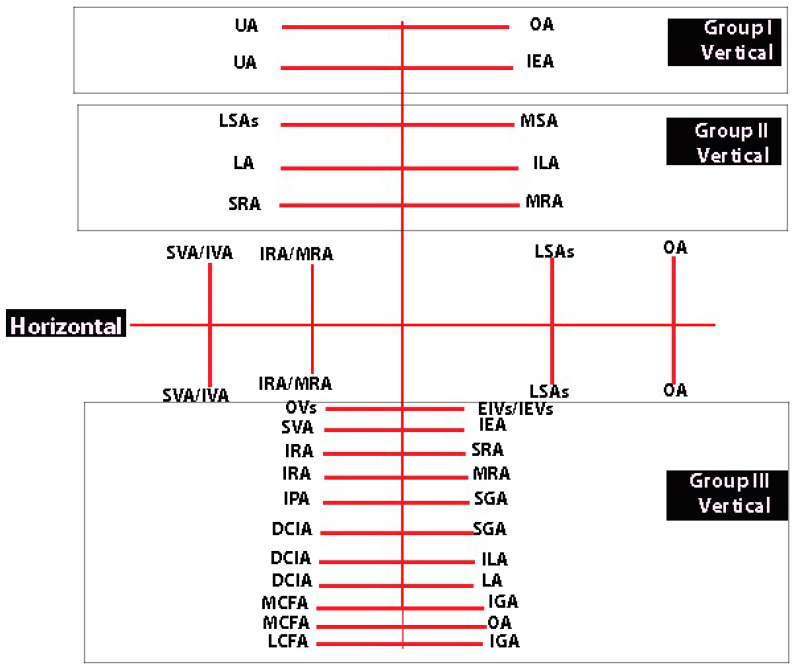
Horizontal and vertical anastomoses of the IIA (author’s own material). UA—uterine artery; OA—obturator artery; IEA—inferior epigastric artery; LSAs—lateral sacral arteries; MSA—median sacral artery; LA—fourth lumbar artery; ILA—iliolumbar artery; SRA—superior rectal artery; MRA—middle rectal artery; SVA—superior vesical artery; IVA—inferior vesical artery; IRA—inferior rectal artery; OVs—obturator vessels; EIVs—external iliac vessels; IEVs—inferior epigastric vessels; IPA—internal pudendal artery; DCIA—deep circumflex iliac artery; MCFA—medial circumflex femoral artery; LCFA- lateral circumflex femoral artery; SGA—superior gluteal artery; IGA—inferior gluteal artery.

The third group comprises anastomoses between most divisions of the large vessels in the pelvis, including the IIA, external iliac, femoral arteries, and divisions of the abdominal aorta (fourth lumbar artery). There is some debate among authors regarding the significance of the divisions from the external iliac artery in IIA ligation, with some suggesting they have no role, while others argue the opposite [5,6,62]. However, it is essential not to overlook anastomoses originating from the external iliac or femoral arteries after IIA ligation. For instance, corona mortis represents a direct vascular communication between the obturator vessels and the external iliac vessels and their divisions (as illustrated in Figure 7) [68]. Additionally, aberrant and accessory obturator vessels could also contribute to the third group of anastomoses. The aberrant obturator vessels can have a caliber higher than 3 mm, and they could represent an additional collateral network [69]. This communication may potentially play a role in preventing postoperative ischemic complications if the IIA is ligated above the posterior division. Moreover, studies show fewer postoperative ischemic complications when the IIA is ligated at its origin [2]. Furthermore, some third-group anastomoses have been observed in cases of aorto-iliac occlusive disease [62]. The inferior rectal artery (branch of the IPA) anastomoses with the ipsilateral and contralateral superior rectal and middle rectal arteries [61]. The IPA also anastomoses with the SGA [17,70]. The terminal branches of the deep circumflex iliac artery anastomose with the terminal branch of the superior gluteal and fourth lumbar arteries, providing a systemic–systemic collateral circulation [62]. The branches of the deep circumflex iliac artery also anastomose with the iliac branch of the iliolumbar artery [1,62]. The medial circumflex femoral artery has two branches—superficial and deep. The latter passes through the pectineus and iliopsoas muscles and reaches the posterior compartment of the thigh, where it anastomoses with the branches of the inferior gluteal and obturator arteries [1,11,17,62]. The lateral circumflex femoral artery has ascending and descending branches. The ascending branch reaches the gluteus medius muscle, where it anastomoses with the branch of the inferior gluteal artery [1,11,17,62]. Braithwaite found that the inferior epigastric artery and the superior vesical artery anastomosed either in the subperitoneal tissue near the urinary bladder or in the wall of the urinary bladder [71]. Shehata investigated the arterial supply of the urinary bladder among 44 infant cadavers and 22 adult cadavers (33 males and 33 females). The author found that the superior vesical artery anastomosed with the inferior epigastric artery only at the extraperitoneal tissue near the urinary bladder. Anastomoses in the wall of the urinary bladder were only observed when the superior vesical artery originated from an aberrant obturator artery [72]. The horizontal and vertical anastomoses of the IIA are shown in Figure 8.

## 10. Fertility and Pregnancy Outcomes following IIA Ligation

IIA ligation appears to be a safe procedure that does not impair subsequent fertility and pregnancy outcomes even in combination with other fertility procedures of the uterus (Bakri balloon and B-Lynch suture [73,74,75,76]. Nizard et al. [10] reported a series of pregnancies following IIA ligation, with normal pregnancy outcomes and no observed infertility among their population. Similarly, Domingo et al. found that bilateral internal iliac artery occlusion (ligation or embolization) effectively controlled postpartal hemorrhage and did not impact fertility, although the absence of uterine revascularization could be a potential concern for future reproduction [77]. Additionally, Papp et al. presented a case of an uneventful pregnancy course with normal fetal and uterine Doppler values in a woman who underwent an IIA ligation two years prior due to severe bleeding after surgical abortion [34]. These findings collectively suggest that IIA ligation, when performed for severe hemorrhage, may have few long-term effects on ovarian function and subsequent fertility.

## 11. Recommendations

1. The procedure should be performed by an experienced gynecologist or oncogynecologist with enhanced knowledge of the anatomy of the pelvis.

2. In some obstetric cases with severe bleeding and hypovolemic shock, the procedure should be completed in less than 10 min.

3. Surgeons should precisely follow the surgical steps.

4. IIA ligation should be performed distal to the origin of the posterior division of the IIA, especially in patients with interrupted blood flow (atherosclerosis, hypertension, diabetes).

5. Bilateral ligation of the IIA is more effective than unilateral. The latter has limited indications (unilateral hematoma).

6. Surgeons should be familiar with perioperative complications and their management.

7. IIA ligation may often be the sole option enabling the preservation of the uterus, a crucial consideration for women with future pregnancy aspirations.

## 12. Conclusions 

IIA ligation is a critical and lifesaving procedure, particularly in cases of severe unexpected hemorrhage in obstetrics and gynecology. The success of this procedure greatly relies on the expertise and skill of the surgeon. Although perioperative complications are rare, some of them (peripheral nerve and buttock ischemia) are associated with long-term recovery risks and negative impacts on the quality of life. The procedure still has applications, especially in developing countries. IIA ligation should be integrated into obstetrics and gynecology training programs. 

## Figures and Tables

**Table 1:** Length of IIA in anatomical studies.

Author	Year	Cadaver Origin	Number of Cadavers or Hemi-Pelvises	Mean Length (cm) (±SD)	Range (cm)
Adachi [19]	1928	Japan	121	44.3 (±1.3)	
Shafiroff et al. [13]	1959	USA	150		1–3 (21%) 3–5 (60%) 5–7 (13%)
Fatu et al. [20]	2006	Romania	100	4.9	2–9
Bleich et al. [18]	2007	USA	54 (right) 54 (left)	26.8 27	0–5.2 0–4.9
Naveen [14]	2011	India	60 (hemi-pelvises)	3.7 (±4.62)	1.3–5.4
Sakthivelavan et al. [16]	2014	India	58	3.7	2.3–7.1
Yuvaraj et al. [21]	2018	India	80 (right) 80 (left)	3.94 (±0.86) 3.61 (±0.63)	2.4–5.4 2.7–4.7

**Table 2:** Anatomical relations of left, right internal iliac artery and ureter.

	Left IIA	Right IIA
Anterior	Parietal peritoneum	Ureter attached to the parietal peritoneum
Posterolateral	External iliac vein, obturator nerve	External iliac vein, obturator nerve
Posteromedial	Internal iliac vein	Internal iliac vein
Lateral	Psoas major muscle	Psoas major muscle
Medial	Ureter	Parietal peritoneum

## Data Availability

The authors declare that all related data are available concerning research by the corresponding author’s email.
